# ImageJ SurfCut: a user-friendly pipeline for high-throughput extraction of cell contours from 3D image stacks

**DOI:** 10.1186/s12915-019-0657-1

**Published:** 2019-05-09

**Authors:** Özer Erguvan, Marion Louveaux, Olivier Hamant, Stéphane Verger

**Affiliations:** 1 Laboratoire de Reproduction et Développement des Plantes, Université de Lyon, UCB Lyon 1, ENS de Lyon, INRA, CNRS, 46 Allée d’Italie, 69364 Lyon Cedex 07, France; 2 Department of Biology, Graduate School of Natural and Applied Sciences, Bursa Uludağ University, Bursa, Turkey; 3 Present Address: Center for Organismal Studies, University of Heidelberg, Im Neuenheimer Feld 230, 69120 Heidelberg, Germany; 4 Present Address: Umeå Plant Science Centre, Department of Forest Genetics and Plant Physiology, Swedish University of Agricultural Sciences, SE-901 83 Umeå, Sweden

**Keywords:** Cell shape, Cell contour, Segmentation, Confocal microscopy, Cell wall, MorphoGraphX, ImageJ, R

## Abstract

**Background:**

Many methods have been developed to quantify cell shape in 2D in tissues. For instance, the analysis of epithelial cells in Drosophila embryogenesis or jigsaw puzzle-shaped pavement cells in plant epidermis has led to the development of numerous quantification methods that are applied to 2D images. However, proper extraction of 2D cell contours from 3D confocal stacks for such analysis can be problematic.

**Results:**

We developed a macro in ImageJ, SurfCut, with the goal to provide a user-friendly pipeline specifically designed to extract epidermal cell contour signals, segment cells in 2D and analyze cell shape. As a reference point, we compared our output to that obtained with MorphoGraphX (MGX). While both methods differ in the approach used to extract the layer of signal, they output comparable results for tissues with shallow curvature, such as pavement cell shape in cotyledon epidermis (as quantified with PaCeQuant). SurfCut was however not appropriate for cell or tissue samples with high curvature, as evidenced by a significant bias in shape and area quantification.

**Conclusion:**

We provide a new ImageJ pipeline, SurfCut, that allows the extraction of cell contours from 3D confocal stacks. SurfCut and MGX have complementary advantages: MGX is well suited for curvy samples and more complex analyses, up to computational cell-based modeling on real templates; SurfCut is well suited for rather flat samples, is simple to use, and has the advantage to be easily automated for batch analysis of images in ImageJ. The combination of these two methods thus provides an ideal suite of tools for cell contour extraction in most biological samples, whether 3D precision or high-throughput analysis is the main priority.

**Electronic supplementary material:**

The online version of this article (10.1186/s12915-019-0657-1) contains supplementary material, which is available to authorized users.

## Background

Cell shape is a primary variable in morphogenesis in all kingdoms, either as a building block for multicellular shape or because cell shape in turn biases the behavior of structural elements (e.g., cytoskeleton) or morphogens. Because plant cells do not migrate, and usually do not go through apoptosis in young tissues, plant morphogenesis primarily relies on cell elongation and cell division. From a geometric perspective, this means that plant morphogenesis mainly depends on the cell growth rate and growth anisotropy [1, 2]. Whether in kinematic analyses (e.g., [3–5]), in functional genetics (e.g., [6, 7]), in cell biology (e.g., [8]), and in computational modeling (e.g., [9]), quantifying cell contours during growth is thus crucial to understand plant development as a whole.

Plant cell shapes depend on internal and external factors. An isolated plant cell is shaped by the balance between turgor pressure and cell wall resistance to turgor. Because turgor pressure is in essence isotropic, any deviation from a spherical shape is determined by the mechanical anisotropy of the cell wall [10]. Typically, wall-less protoplasts are spherical. Cellulose microfibrils are classically thought to play a load-bearing role here, and their alignment supports the mechanical anisotropy of the wall. In fact, when cellulose deposition is impaired, cells also tend to become spherical, as in protoplasts [11, 12]. Beyond the wall properties, the mechanical balance operating in plant cells also depends on cell shape. Typically, when they are still growing, larger cells are more susceptible to wall failure than smaller cells [9].

In tissues, cell shape is also constrained by the presence of adjacent cells, through packing and adhesion at the middle lamella. This explains why most plant cells in fully adhesive tissues have a brick shape (e.g., hypocotyl cells). When cell-cell adhesion is artificially affected, cells can round up [13]. Similarly, when cell-cell adhesion is less prominent naturally, cells can also round up or exhibit irregular shapes, as in the leaf mesophyll and spongy parenchyma for instance. Yet, even when cells are fully adhering to one another, they can still display wavy cell walls. This is notably the case for puzzle-shaped pavement cells in most leaf epidermises.

Extracting cell contours can also help understand the mechanics behind shape changes. For instance, in jigsaw puzzle-shaped pavement cells, the presence of alternating convex and concave walls has been associated with differences in the mechanical and chemical properties along and across anticlinal walls [14]. Conversely, the shape of such cells prescribes a tensile stress pattern at the outer wall [15], opening the way for mechanical and chemical interplays between the different cell sides. The detection and quantification of cracks, and the resulting cell deformation, in mutants with adhesion defects can also inform on the tensile stress pattern [16]. Incidentally, such analysis confirmed the presence of directional tensile stress in pavement cell neck regions [17]. This also has implications for molecular factors. The role of RhoGTPase is, for instance, well established for pavement cells [18].

Depending on the type of cell shape, different parameters can be extracted, such as length, width, outer wall area, or volume. As a first solution, cells can be approximated as ellipsoids, meaning that a minor and major axis can be calculated. However, as cells typically have more complex shapes than cuboid or ellipsoid, such simplification can prove problematic. This approach for instance is not appropriate for cells with wavy walls, such as pavement cells. A number of tools have been developed to extract more accurate geometrical representations of such cells and, by extension, of any cells. For instance, by extracting the cell contour, solidity or circularity can be deduced. Lobe number and lobe size have also been measured from such contour extraction [18]. A recently developed ImageJ plugin allows the extraction of 27 geometrical parameters that are relevant to plant cell shapes in all their diversity, from cell contours [19]. Fourier transform-based protocols have also been successfully used to obtain the main descriptors of pavement cells automatically [20].

Yet, all these methods require good quality 2D images of cells contours, which is not always easy to obtain from 3D stacks. Unfortunately, very few dedicated tools are available for this task. The Python-based MerryProj tool [21] was developed for this purpose but is not maintained anymore. A tool called SurfaceProject, part of the Simplant library [22], was developed based on a different principle but for a similar purpose. However, it requires manual processing of each image. Very recently, the ImageJ plugin LSM-W^2^ was also introduced [23]. One of the tools developed within this plugin allows the creation of virtual cuts through 3D stacks. Unfortunately, it can only be used for images in the “lsm” Zeiss proprietary confocal microscopy image format, and the method relies on assumptions that can make it less versatile for different types of samples. Another method called “Smooth 2D manifold extraction from 3D image Stack” has also been recently introduced [24]. This method provides a very robust approach to extract a 2D layer of signal preserving the local spatial relationship of the stack content. However, this is a parameter-free method, which does not allow the user to precisely specify which layer of signal will be extracted. The three-dimensional image analysis software MorphoGraphX (MGX) [25] is the most versatile and accurate tool available at the moment among image analysis freewares to extract 2D cell contours. Nevertheless, the version currently available online for download requires specific hardware, and it can have a steep learning curve. Here, we introduce a new high-throughput method dedicated to cell contour extraction from 3D stacks (SurfCut) and compare it with MGX. We discuss the associated advantages and limitations.

## Methods

### Plant material and growth conditions

*Arabidopsis thaliana* wild-type Col-0 and the microtubule reporter line *GFP-MBD* (WS-4, [26] were used in this study. Seeds were cold treated for 48 h to synchronize germination. Plants were then grown in a phytotron at 20 °C, in a 16-h light/8-h dark cycle on solid Murashige and Skoog medium (MS medium, Duchefa, Haarlem, the Netherlands) with 0.8% agar, 1% sucrose, and no vitamin. Seedling age was counted from the start of light exposure.

### Confocal microscopy

Cell contour staining was performed by staining the cell wall with propidium iodide (PI). Plants were immersed in 0.2 mg/ml propidium iodide (PI, Sigma-Aldrich) for 10 min and washed with water prior to imaging. For imaging, samples were either placed on a solid agar medium and immersed in water or placed between a glass slide and coverslip separated by 400 μm spacers to prevent tissue crushing. Images were acquired using a Leica TCS SP8 confocal microscope, equipped with a water immersion objective (HCX IRAPO L × 25/0.95 W). PI excitation was performed using a 552-nm solid-state laser, and fluorescence was detected at 600–650 nm. GFP excitation was performed using a 488-nm solid-state laser, and fluorescence was detected at 495–535 nm. Stacks of 1024 × 1024 pixels (pixel size of 0.363 × 0.363 μm) optical section were generated with a *Z*-interval of 0.5 μm.

Note that for both methods to work, the acquired signal must be strong and continuous enough at the edge of the sample in order for the signal to be detected and segmented from the background noise by a simple conversion to a binary image. For instance, staining of the membranes, or the cell walls in the case of plants, is usually ideal. A more heterogeneous signal such as the cortical microtubules (as in the case of the hypocotyl here; Fig. 4) can also be used, given that it covers enough of the surface of the sample and does not leave large signal holes. It is also important to avoid the presence of artifacts, e.g., from stained cell debris or bacteria at the surface of the sample.

### 2D cell contour extraction with MGX

**Fig. 1:**
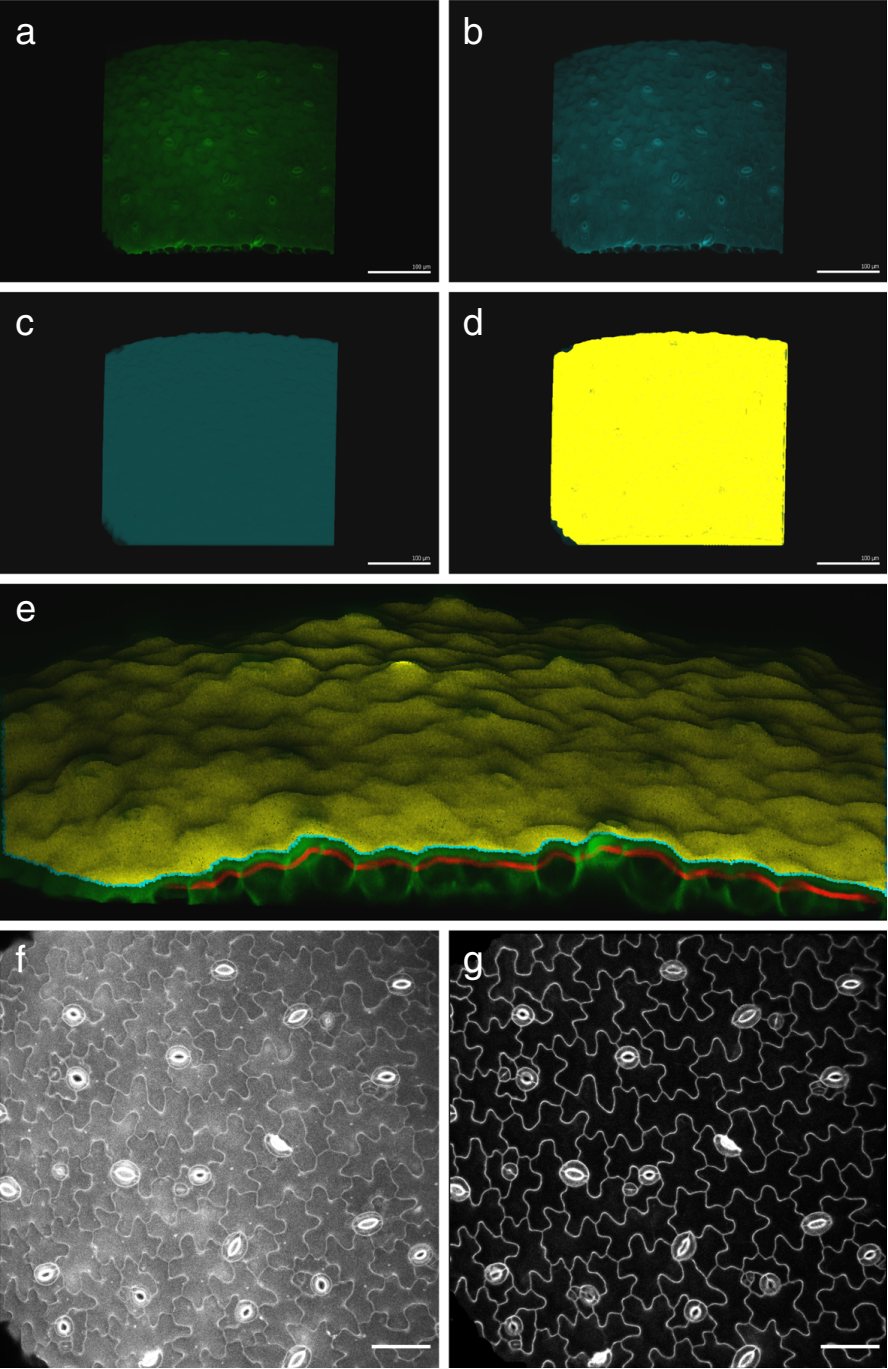
2D cell contour extraction with MGX. **a** Original confocal stack opened in MGX: cotyledon pavement cells of *A. thaliana*, stained with propidium iodide. **b** Confocal stack from **a** after a Gaussian Blur (0.3-pixel radius). **c** Detected surface using the Edge Detect process. **d** Mesh created using a 5-μm Marching Cubes Surface process, then smoothing one time and subdivision one time. **e** Original confocal stack (green), surface’s mesh created in MGX (yellow), and the 2-μm-thick layer of signal cropped at a distance of 6 (top) to 8 (bottom) μm from the surface in MGX (red). The view of the sample is tilted to allow better visualization of the mesh, the original signal, and the cropped stack. **f** Grayscale Z-projections (maximal intensity, in Fiji) of the entire original confocal stack (from **a**). **g** Z-projections (maximal intensity, in Fiji) of the 2-μm-thick layer of signal extracted, in red in **e**. Scale bar in **a**–**d** is 100 μm and in **f**–**g** is 50 μm

Confocal stacks were opened with the open source software MorphoGraphX (www.morphographx.org; Fig. 1a). In order for the process to work properly, the first slice of the stack should be the top of the outer side or the top of the surface of the sample relative to which you want to extract the signal. Then, for each confocal Z-stack, de-noising of the raw signal was performed using the “Gaussian Blur Stack” process with a 0.3-pixel radius (in MGX, *Process > Stack > Filters > Gaussian_Blur_Stack*; Fig. 1b). The edges of the confocal signal were obtained using the “edge detect” process (*Process > Stack > Morphology > Edge Detect*; threshold 10000, multiplier 2, adaptative factor for threshold 0.3, fill value 15000; Fig. 1c). A mesh was created using the “Marching Cubes Surface” process (*Process > Mesh > Creation > Marching_Cubes_Surface*; cube size 5 μm, threshold 5000; Fig. 1d). The mesh was then smoothed and subdivided using the “Smooth Mesh” and “Subdivide” processes (*Process > Mesh > Structure > Smooth_Mesh* and *Process > Mesh > Structure > Subdivide*; Fig. 1d). The original stack was then cropped using the “Annihilate” process with a minimal distance of 6 μm and a maximal distance 8 μm from the mesh (*Process > Stack > Mesh_Interaction > Annihilate*; Fig. 1e). The cropped stack was then saved as a TIFF (*Stack > Stack1 > Work > Save*). Note that the exact values for each parameter depend on initial raw data.

2D cell contour images were then generated using Fiji (https://fiji.sc/; [27]). The image type was first changed to 8 bit (in Fiji, *Image > Type > 8-bit*), and the stack was projected in 2D using the “Z Projection-Max Intensity” function (*Image > Stacks > Z Projection*; Fig. 1g).

### 2D cell contour extraction with SurfCut

**Fig. 2:**
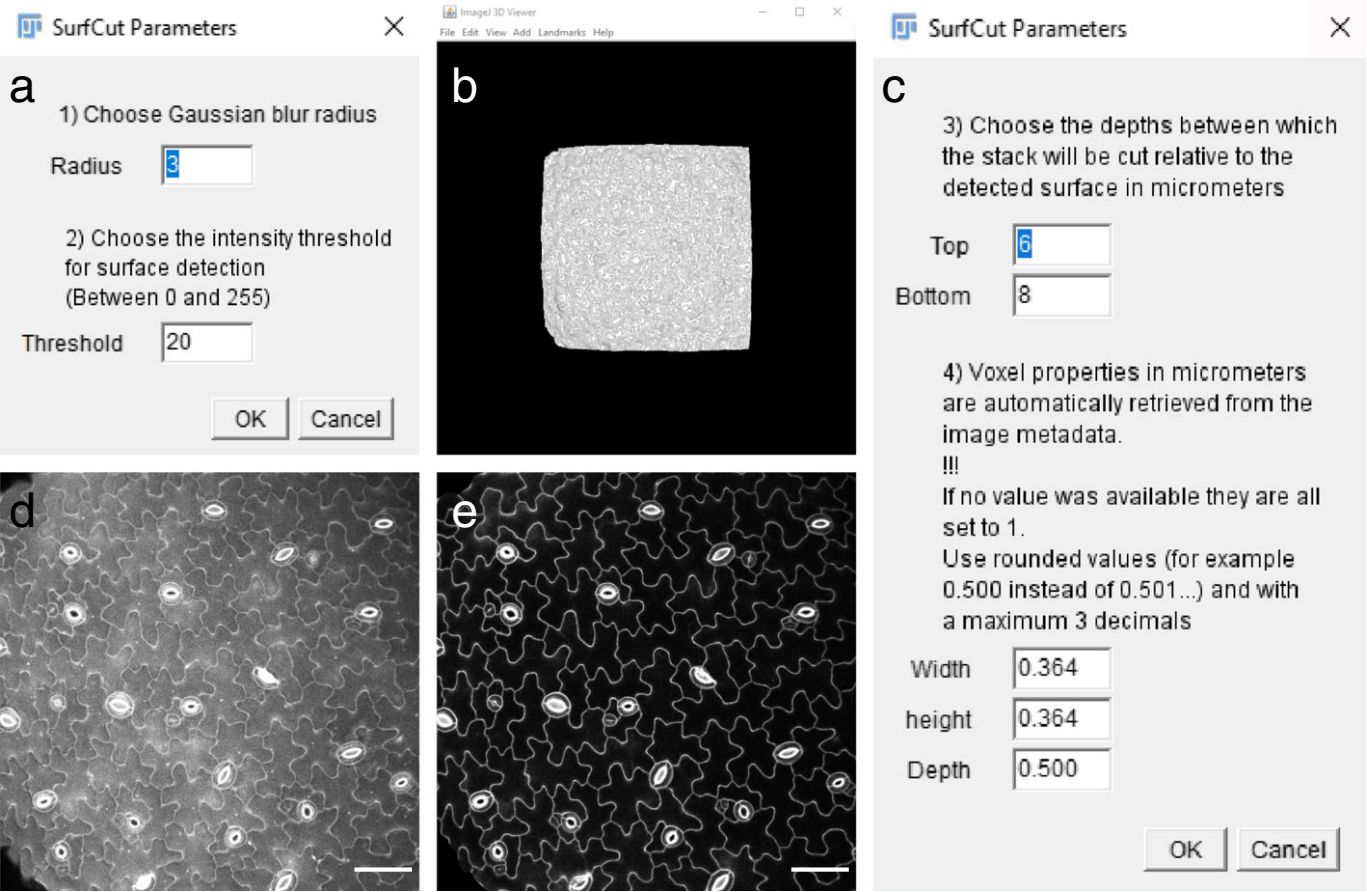
2D cell contour extraction with the Fiji SurfCut macro. **a**–**c** Illustration of the procedure used in order to determine the appropriate parameters for the cell contour extraction using the SurfCut “Calibrate” mode. **a** The macro first proposes to choose the radius to be used for the Gaussian Blur filter as well as the threshold for the signal binary conversion. This step is equivalent to the edge detect process of MGX. **b** The output of **a** can be visualized in the 3D viewer of Fiji to inspect it. **c** The desired depth of cropping can then be chosen. The voxel properties are automatically retrieved from the metadata but can be further adjusted. **d** Grayscale Z-projections (maximal intensity) of the original confocal stack (same as Fig. 1f). **e** Z-projection (maximal intensity) of the stack cropped with SurfCut. Scale bar is 50 μm

We developed a simple ImageJ macro that we named SurfCut (note that this is not related to the image segmentation method with the same name). The scripts as well as a more detailed step by step user guide are available at https://github.com/sverger/SurfCut (Zenodo DOI:10.5281/zenodo.2635737 [28]) and in Additional files 1 and 2 [29, 30]. The macro has two modes: (1) “Calibrate,” to manually find the proper settings for the cell contour extraction; this mode can also be used to process samples manually one by one (Fig. 2); (2) “Batch,” to run batch cell contour extraction on series of equivalent Z-stacks, using appropriate parameters as determined with the “Calibrate” mode. In order to run the macro, the script should be opened in Fiji (*Plugins > Macros > Run…*, and then select the “SurfCut.ijm” file, or drag and drop the “SurfCut.ijm” file in Fiji and click “Run”).

In this macro, the cell contour extraction is done using a succession of classical ImageJ functions. The first slice of the stack should be the top surface of the sample in order for the process to work properly. The stack is first converted to 8 bit. De-noising of the raw signal is then performed using the “Gaussian Blur” function. The signal is then binarized using the “Threshold” function, and an equivalent of the “edge detect” process from MGX is performed. This is an important step in order to create a “filled” binary object encompassing the whole sample signal. The binary conversion with a simple threshold would leave “holes” within the object due to the absence of signal inside the cells, and such holes would be problematic in the following processing steps, when the object is used as a mask to crop the signal. To perform such “edge detect” step, each slice from the binarized stack, starting from the top slice, is successively projected (Z-project) with the upper slices in the binarized stack. In other words, a new stack is created in which the first slice is simply the first (top) slice from the binarized stack, the second slice is a projection of the first and second slice, the third slice is a projection of the first to the third slice, etc. This ultimately creates a new binary stack in which all the binary signals detected in the upper slices appears projected down on the lower slices, effectively filling the holes in the binary object. This new stack is then used as a mask shifted in the Z direction, to subtract the signal from the original stack above and below the chosen values depending on the desired depth of signal extraction. The cropped stack is finally projected along the *z*-axis using the maximal fluorescence intensity in order to obtain a 2D image. The values of the parameters for each of the functions need to be determined with the “Calibrate” mode (Fig. 2 and Additional file 2).

### Pavement cell analysis with PaCeQuant

2D cell contour images were created with both MGX and SurfCut methods. The PaCeQuant tool [18] from in the MiToBo plugin in Fiji was used to segment the output images, generate the ROIs for each cell, and quantify the cell shape parameters. A threshold of 2500 pixels was used to filter out the smaller cells during the segmentation step in order to exclude the guard cells. To compare the accuracy of the surface extraction in both methods, the acquired datasets were further analyzed using the R package PaCeQuantAna [18].

### Cell size quantification in 2.5D with MGX

We used MGX to quantify the cell surface area in 2.5D following the step-by-step user manual associated with the software (MGXUserManual steps 3, 4, 6, 7, 8, and 9) [25].

### Assessment of cell and tissue curvature bias on cell size quantification

We quantified cell size using either 2D SurfCut/PaCeQuant (cell contours extracted with SurfCut and cell size measured with PaCeQuant) or 2.5D MGX (see above) methods and calculated the error for each segmented cells, knowing that MGX fully accounts for cell and tissue curvature while SurfCut does not. This percentage difference is calculated using (1) 2.5D MGX and (2) 2D SurfCut cell area as such: (((1)–(2))/(1)) × 100. The 2D SurfCut/PaCeQuant cell area was represented as a heatmap in Fiji, while the 2.5D MGX cell area as well as the difference values was represented as a heatmap in MGX using the mesh defined during the 2.5D MGX analysis. We also measured the average angle of each cell files relative to the top view on a transverse section of the hypocotyl using the angle tool in Fiji. For each cell files, we drew a line between the two outer cell wall cell-to-cell junctions and measure the angle between this line and the bottom line of the image. This operation was repeated four to five times per cell file along the hypocotyl to get an average cell file angle.

### Statistical analysis and data visualization

Cell shape quantifications obtained with both methods were statistically compared in R [31] and visualized with boxplots using ggplot2 [32]. Because some of our data had non-normal distributions, we used two-sided Wilcoxon rank-sum tests for the comparisons.

## Results and discussion

### 2D cell contour extraction from 3D samples with MGX and SurfCut

Here, we report a new method (SurfCut) to extract cell contours or specific thin layers of a signal at a distance from the surface of samples in 3D confocal stacks. The goal is notably to obtain the cell contours of the epidermal layer in a tissue, by extracting the signal from the epidermal anticlinal walls only. We compare these new methods with the 3D image analysis software MorphoGraphX (MGX) [25].

In MGX, a 3D triangle mesh is created from a confocal stack, corresponding to the edges of the sample’s signal, and notably the surface of the sample (see the “Methods” section and Fig. 1a–d). This mesh can then be used to crop the raw confocal signal at a chosen distance from the sample’s surface to extract a thin layer of signal (Fig. 1e).

**Fig. 3:**
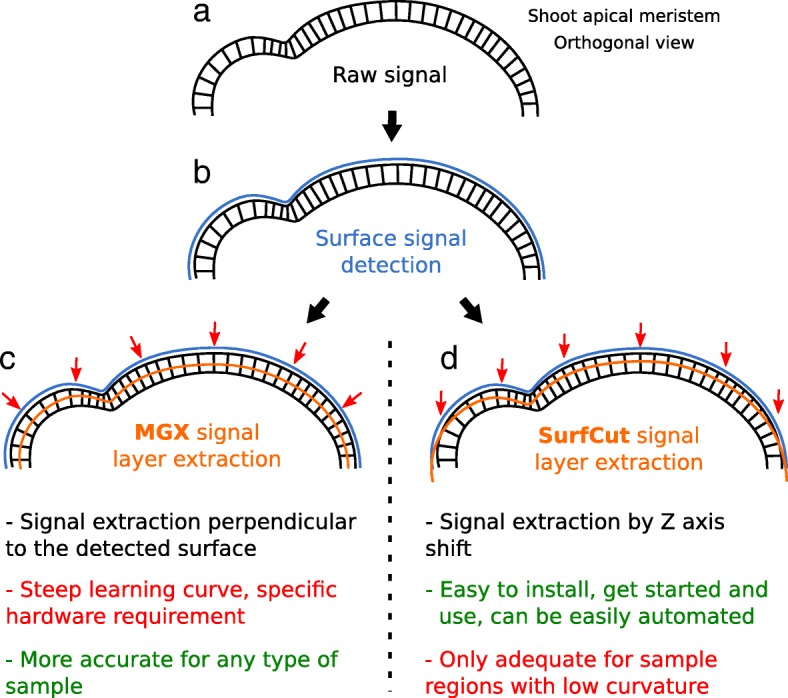
MGX vs. SurfCut signal layer extraction: principles, pros, and cons. Illustration of the principles of the signal layer extraction for MGX and SurfCut on the highly curved shoot apical meristem. **a** Schematic representation of the orthogonal view of the shoot apical meristem. Only the epidermal cell layer is drawn. **b** The surface signal is detected (blue) from the raw 3D confocal signal (black) and used as a basis to crop the raw signal at a given depth (orange), either **c** perpendicular to the surface (MGX) or **d** in the *z*-axis (SurfCut)

We developed an ImageJ macro with the aim to obtain a rather equivalent signal layer output, in a simpler, but less versatile, setup. In this case, instead of creating a mesh, the binarized “filled” signal of the sample is used as a mask to crop the raw confocal signal at a chosen *Z*-depth relative to the surface (and thus not exactly perpendicular to the surface as for MGX; see the “Methods” section and Figs. 2 and 3).

**Fig. 4:**
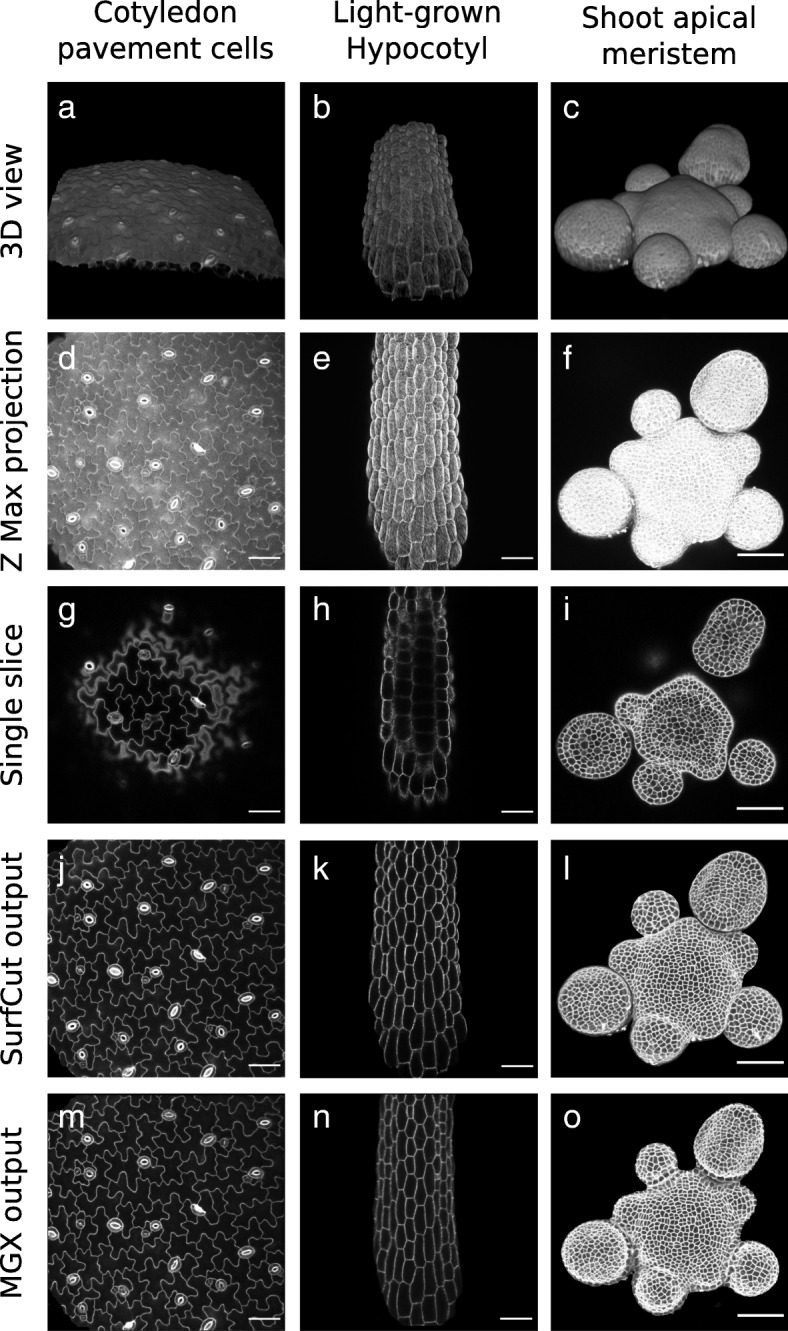
Examples of SurfCut and MGX cell contour extraction in various sample types. **a**, **d**, **g**, **j**, **m** Propidium iodide-stained cotyledon pavement cells. **b**, **e**, **h**, **k**, **n** light-grown hypocotyl expressing the *GFP-MDB* reporter line. **c**, **f**, **i**, **l**, **o** propidium iodide-stained shoot apical meristem. **a**–**c** 3D views of the samples. **d**–**f** Maximal intensity projection. **g**–**i** Single slice through the sample. **j**–**l** SurfCut output. **m**–**o** MGX output. Panel **d** is the same as Figs. 1 f and Fig. 2d. Panels **j** and **m** are the same as Fig. 2e and Fig. 1g, respectively. Scale bar is 50 μm

As a proof of concept for our cell contour extraction methods, we acquired 3D confocal Z-stacks from three different non-fully flat samples: cotyledon pavement cells (relatively flat, Fig. 4a), light-grown hypocotyls (curved along one axis, Fig. 4b), and shoot apical meristem (highly curved and complex, Fig. 4c). Performing a classical maximal intensity Z projection on these stacks generates 2D images in which cell contours are almost impossible to identify or segment because multiple cell layers and periclinal walls overlap (Fig. 4d–f). Furthermore, in these samples, as in almost any 3D confocal stack of such non-fully flat samples, taking a single slice through the stack does not allow to obtain the cell contours of a single cell layer for the whole image (Fig. 4g–i).

We next applied our signal layer extraction methods on these samples. Both methods seem to yield good quality, and rather similar, 2D images of cell contours from the epidermal layer (Fig. 4j–o). This is in principle very close to reality for the relatively flat samples such as cotyledon pavement cells. Indeed, due to the geometry of this type of sample, both procedures should produce roughly the same output. In contrast, a closer look in the case of the hypocotyl and the shoot apical meristem reveals visual differences. For instance, the output in Fig. 4k (SurfCut) is wider than that in Fig. 4n (MGX), and this is directly due to the difference in signal extraction method (see Fig. 3). In principle, the method using MGX is more accurate, especially on very curved samples, and the MGX environment allows many more analyses. However, if cell contour extraction is the sole priority and the sample geometry is not too complex (see limitations of the 2D SurfCut method in Fig. 6), the current version of MGX still has the drawback to require a specific graphics card and to be rather complex to automatize for batch analyses. SurfCut is less accurate because it does not crop the signal perpendicular to its surface but simply in the Z direction. Therefore, as exemplified above, the associated error can become important for samples with high curvature. In contrast to MGX, the SurfCut-based workflow has the advantage to be much simpler to use and outputs rather similar results as MGX in many cases, the main limitation being the curvature of the sample (see Figs. 3 and 6). In addition, it is much easier for a biologist with little or no knowledge of programming to automatize in order to run in batch on multiple samples without manual processing (see Additional file 2). Last, SurfCut can be run with Fiji, a widely-used image processing software that does not require a specific graphics card.

### Quantitative comparison of MGX and SurfCut cell contour extraction with cotyledon pavement cell shape analysis

Because we found that the two methods output qualitatively rather similar results in the flat regions of our samples, we decided to compare the methods in a more quantitative way. We decided to first focus on cotyledon pavement cells because the output differences were hardly noticeable by eye, contrary to the hypocotyl and the shoot apical meristem. In order to test this, we used a set of eight 3D confocal stacks of cotyledon pavement cells that we processed with both methods to obtain 8 2D images of cell contours as described above. We then used the ImageJ plugin “PaCeQuant” [19] to obtain the corresponding cell shape descriptors. As mentioned earlier, this plugin carries out very efficient cell segmentation from 2D images and can compute 27 different shape features based on global, contour-based, skeleton-based, and PC-specific features such as area, perimeter, length, or width.

**Fig. 5:**
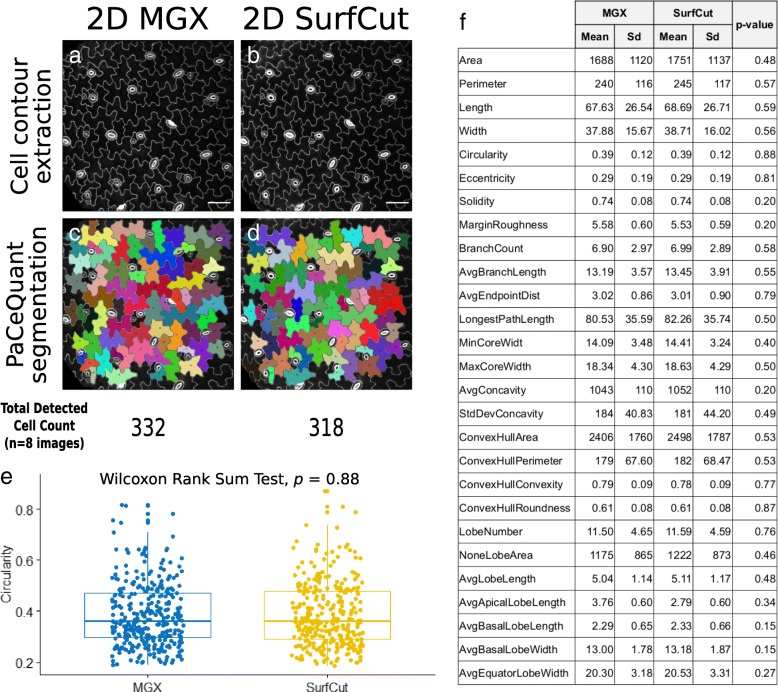
Quantitative comparison of 2D MGX and 2D SurfCut output. **a** Z-projections of cell contour images extracted either with MGX (similar to Figs. 1g and 4m) or **b** with SurfCut (similar to Figs. 2e and 4j) from the same original stack. **c**–**d** Same images as in **a** and **b**, respectively, segmented with PaCeQuant. Below is the total number of cell detected in the eight samples for each method. **e** Boxplot of the circularity values, representing each data point (each point corresponding to one cell) and their distribution for both cell contour extraction method. Wilcoxon rank-sum test of the comparison between both methods output a *p* value of 0.88. **f** Table reporting for each PaCeQuant shape parameter, the mean and standard deviation (sd) for both the MGX and SurfCut method, and the *p* value of the Wilcoxon rank-sum test comparing the two methods. Scale bars 50 μm

First, we compared the number of segmented cells after cell contour extraction using both methods, as well as with manual counting. From the 8 images, we manually counted 352 cells, while PaCeQuant segmentation following the MGX-based method allowed us to detect a total of 332 cells (Fig. 5a, c, e), and PaCeQuant segmentation following the SurfCut macro allowed us to detect a total of 318 cells (Fig. 5b, d, e). Compared to the manual count, this represents 94% of detected cells for MGX and 90% for SurfCut. Thus, both methods output rather similar results. However, both methods did not seem to allow 100% of cell detection. After closer examination, we could identify that most of the difference with the manual counting results from the filtering out of small cell (< 2500 pixels of the area) in the PaCeQuant segmentation algorithm, which is meant to exclude the guard cells from the analysis. This represents about 8–9% of the cells manually counted in the images. We could furthermore observe few cases of over-segmentation as well as segmentations of “incomplete cells” in the case of the MGX output. Incomplete cells are cells located at the border of the image and for which part of the cell surface is missing. These cells are in principle filtered out of the analysis by PaCeQuant to avoid bias. The MGX extraction method tends to create an artificial border (of different pixel intensity) for this type of cells because of a black margin artificially created around the image. This black margin originates from the signal extraction method: the surface being relatively convex, the signal extracted perpendicular to the surface is therefore slightly smaller in width (see Fig. 3 and Fig. 4k, n). This relative over-segmentation surprisingly makes the 2D MGX method less accurate for these cells.

Next, we tested whether these differences in segmented cell number would affect the distribution of pavement cell descriptors. Among the features that can be quantified using the PaCeQuant plugin, circularity indicates how similar a cell shape is to a circle (the maximum value of 1 corresponds to a perfect circle). In our sample set, we found that the circularity of the cell contours extracted with the MGX method was 0.3868 ± 0.1233 and for those extracted with the SurfCut script was 0.3856 ± 0.1247 (Fig. 5e), revealing no statistical differences between the two tested populations (Wilcoxon rank-sum test *p* value = 0.88). To push the analysis further, we also compared each of the 27 descriptors available with PaCeQuant (Fig. 5f). Despite more noticeable differences for some parameters, this comparison could not reveal any statistical differences between the two cell contour extraction methods (Fig. 5f). Altogether, our analysis suggests that in the case of the cotyledon pavement cells, despite relatively minor qualitative differences, both cell contour extraction methods are valid. Furthermore, it reveals that SurfCut is well suited for high-throughput pre-processing of 3D confocal stacks for pavement cells shape quantifications.

### Quantitative comparison of 2.5D MGX and 2D SurfCut in samples with complex 3D geometry

**Fig. 6:**
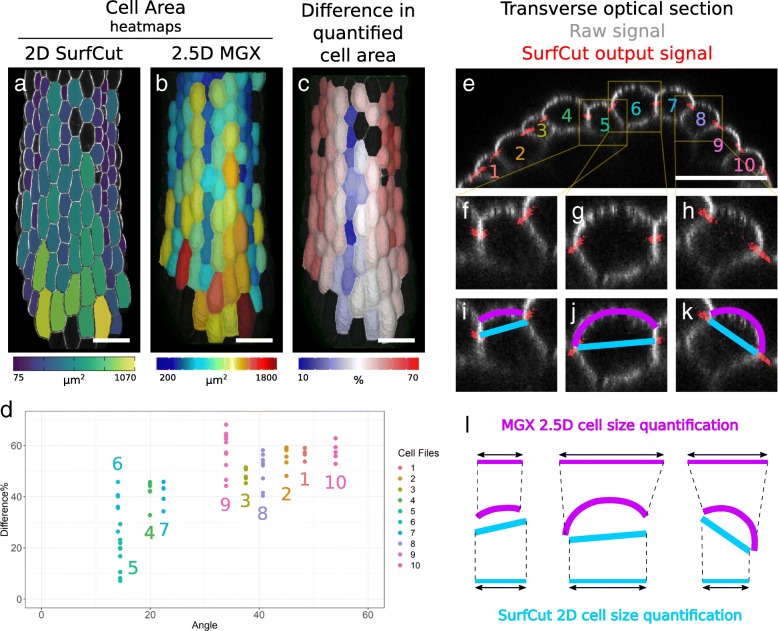
Quantitative comparison of 2.5D MGX and 2D SurfCut output. **a**–**c** Quantitative comparison of cell area on light-grown hypocotyl (same raw data as in Fig. 4b). **a** 2D analysis of cell area: cell contour extracted with SurfCut, cells segmented, and cell size quantified with PaCeQuant. **b** 2.5D analysis of cell area in the same light-grown hypocotyl using MGX. **c** Visual representation of the difference of cell size quantification between the two methods. Heatmaps in **a** and **b** represent the distribution of quantified cell size (μm^2^) while in **c**, it represents the difference in quantification between both methods. Positive values in **c** correspond to cases in which cell area quantified in 2.5D MGX is higher than in 2D SurfCut. **d** Scatter plot of the difference in cell size quantification relative to the angles of cell files in the tissue. The cells in the hypocotyl are organized in cell files. Each cell file is highlighted by a unique color and number corresponding to the colors and numbers in **e**. Here, each cell file was assigned a single average angle (see the “Methods” section). Cells “facing” the top view have a low angle. **e**–**k** Transverse optical section of the light-grown hypocotyl shown in **a**–**c**, showing in greyscale the raw confocal signal and in red the thin layer of signal extracted using SurfCut. **f**–**l** Close-ups (insets in **e**) highlighting one case in which the bias is almost completely absent (cell file 5 (**f**, **i**)) and two cases in which cell area measurement is biased (cell files 6 (**g**, **j**) and 8 (**h**, **k**)). The magenta and cyan lines in **i**–**l** are 1D representations of the MGX (magenta) and SurfCut/PaCeQuant (cyan) cell area quantification. **l** Schematic representation and explanation of the induced bias. As opposed to 2D SurfCut/PaCeQuant, cell area measurement in 2.5D with MGX fully accounts for the tissue- and cell-level curvature. Scale bars 50 μm

Although SurfCut in combination with PaCeQuant allows for a simple and high-throughput cell shape analysis, one of the main limitations of our method is that it does not take into account the curvature of the tissue or the cells. In order to quantify this limitation, and better inform the users on the potential bias, we decided to compare a 2.5D analysis of the hypocotyl sample in MGX with the 2D analysis in SurfCut, focusing on cell size quantification. To do so, we first quantified cell size in 2D using the SurfCut output and PaCeQuant segmentation and cell area quantification, and in 2.5D using MGX. Both outputs are represented as heatmaps of the cell area (Fig. 6a, b). In all cases, and as expected, 2.5D MGX cell area quantification provided higher values than 2D SurfCut/PaCeQuant. To better visualize the difference in cell size quantification between the two methods, we also generated a heatmap of the percentage of difference (Fig. 6c). Cells which have a higher difference in cell area quantification are in warm colors while cells with low difference are in colder colors (Fig. 6c). The heatmap highlights a bias of cell size quantification for the cells which are on the side of the hypocotyl. To further quantify this bias, we also measured the average angle of the top walls in the different cell files relative to the top view of the stack. This measurement is taken in the transverse axis of the hypocotyl (Fig. 6e) from one top cell wall junction to the other and is averaged per cell file (thus, there is only one angle value per cell file). We then plotted the difference in cell size quantification relative to the average cell surface angle (Fig. 6d). We found a trend of increasing difference in cell size quantification with increasing cell angle, but the correlation appears noisy. For instance, for cell files 5 and 6 which both have a low average angle (Fig. 6d–g, i, j), the difference in cell size varies from 10 to 30% and 25 to 35%, respectively (Fig. 6d). This is due to the additional effect of single-cell curvature (Fig. 6e–l). Indeed, the cells in the hypocotyl can be very “bumpy,” and this varies between cell files (Fig. 6e–h). In 2.5D MGX, the cell surface quantification takes fully into account this curvature, which in some cases further increases the difference in cell size quantification. In Fig. 6e–l, we further highlight cell file 5 (Fig. 6e, f, i, l) in which there is very little to no significant bias, cell file 6 (Fig. 6e, g, j, l) in which only cell curvature significantly biases the measurement, and cell file 8 (Fig. 6e, h, k, l) in which both tissue and cell curvature bias the measurements. Such cell-level bias could also exist for the pavement cell analysis, but the global curvature of the cells as well as the variation of curvature between different cells is much lower than in the hypocotyl, and depending on the needs of the experiment, this bias can be considered negligible. On the other hand, in the example of the shoot apical meristem, the single-cell curvature is very low while the global tissue curvature is high, leaving mostly the tissue curvature bias for cell size quantification.

Overall, our pipeline combining SurfCut and PaCeQuant is appropriate for the quantification of cell shape and size in samples with a low tissue and cell curvature, such as the cotyledon epidermis, but not for more complex samples such as the hypocotyl and the shoot apical meristem.

## Conclusions

We developed SurfCut, a user-friendly ImageJ tool amenable to extract cell contours from 3D image stacks. In principle, this tool may be used on any 3D stack (e.g., confocal or light sheet microscopy) originating from either animal, fungi, or plant systems. When compared to MGX, SurfCut requires less expertise and no specific hardware (graphics card). SurfCut is particularly well suited for tissues with a low curvature and can easily be used in batch processes, meaning that high-throughput cell contour extractions can be performed. Notably, we demonstrate here that SurfCut is very well suited for high-throughput pavement cell contour extraction and further quantification. However, SurfCut does not fully account for the 3D shape of the sample such that significant bias can be introduced when analyzing very curvy samples, as demonstrated here with the quantification of the hypocotyl epidermis cell sizes (Fig. 6). This should be carefully considered by the user to determine whether more advanced software such as MGX is more suited for a given analysis. In addition, SurfCut can also be used to extract other types of signals, such as cortical microtubules, allowing a suppression of the background noise coming from the signal below. We could for instance combine a high-throughput cell contour as well as cortical microtubule signal extraction and use the cell contours for automated cell segmentation and generation of ROI within which cortical microtubule arrays were automatically analyzed using an automated version of FibrilTool (Additional file 2, [33]), overall yielding a very high-throughput cortical microtubule analysis in many samples. Finally, SurfCut can be a very useful tool for the 2D representation (from image-based screening protocols to publication figures) of 3D confocal data in which overlapping signal from different depths in the stack hinders the visualization of signal or structures of interest.

SurfCut has some similarities with the Python-based MerryProj tool [21], although they work in a different way. MerryProj used local transparency masks of various intensities around the confocal signal in order to render an image of the surface signal. In contrast to MGX or MerryProj, SurfCut is an ImageJ plugin and not a standalone software, and it does not require specific hardware. The SurfaceProject and LSM-W^2^ [22, 23] ImageJ plugins also represent very good alternatives. For instance, SurfaceProject can be used to extract a layer of signal independently from the surface of the signal, by manually placing points within the 3D stack. These points are then used to define a surface for signal extraction. It is thus more versatile than SurfCut for some cases but requires extensive manual processing of each image. On the other hand, the “2D virtual cut” tool of LSM-W^2^ is more similar to SurfCut. It uses a mask derived from the surface topology to create a virtual cut through the stack. However, it relies on the assumption that the maximum of fluorescence signal intensity is concentrated at the surface of the sample, which is not always the case. In addition, it can only be used for images in the “lsm” Zeiss proprietary confocal microscopy image format.

Overall, despite some limitations, SurfCut brings ease of use and high-throughput capacities, while offering complementary advantages with other existing methods and more advanced software such as MGX, for most applications in cell and developmental biology.

## Additional files

Additional file 1: SurfCut macro. Text file containing the source code of the Fiji macro SurfCut. (TXT 14 kb)

Additional file 2: SurfCut user guide. Step-by-step user guide for the Fiji macro SurfCut. Also presents an example of the use of SurfCut (semi-automated high-throughput cortical microtubule array analysis). (PDF 2277 kb)
