# Supplementary material for: ImageJ SurfCut: a user-friendly pipeline for high-throughput extraction of cell contours from 3D image stacks
Source: BMC Biol. 2019 May 9;17:38. doi: 10.1186/s12915-019-0657-1 (PMC6509810; doi:10.1186/s12915-019-0657-1)
Supplement: Supplementary file 2 — SurfCut user guide. Step-by-step user guide for the Fiji macro SurfCut. Also presents an example of the use of SurfCut (semi-automated high-throughput cortical microtubule array analysis). (PDF 2277 kb) [file 12915_2019_657_MOESM2_ESM.pdf]

Step by step user guide for SurfCut signal layer extraction

Prerequisites:

- Fiji (https://fiji.sc).

- The "SurfCut.ijm" macro file (https://github.com/sverger/SurfCut).

- Data: 3D confocal stacks in .tif format, in which the top of the stack should also be the top of the sample (example data available at https://github.com/sverger/SurfCut/test_File and http://doi.org/10.5281/zenodo.2577053).

Procedure

A. Image acquisition:

Our image analysis pipeline was developed to extract cell contours or specific layers of signal in confocal images of plant samples, but can in principle be used on any 3D fluorescence microscopy stack (e.g. confocal or light sheet microscopy) originating from either animal, fungi or plant systems, stained or expressing a fluorescent reporter highlighting the cell contours (typically, a protein at the plasma membrane). For a better-quality output, it is recommended to use a Z interval of maximum 1 µm.

B. Starting the SurfCut macro:

To manually determine the proper settings for the signal layer extraction.

1. Start Fiji.

2. Run the macro: In Fiji go to *"Plugins"* and then *"Macros"* and *"Run..."*.

   This will open a navigation window from which you can select the "SurfCut.ijm" file, wherever you have downloaded it. Then click on "open".

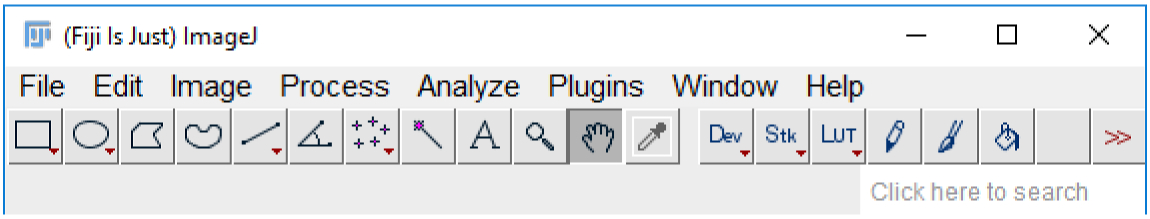

   Note that alternatively you can drag and drop the "SurfCut.ijm" file on the Fiji window, and click "run" in the new window.

3. The macro will start and display the window below.

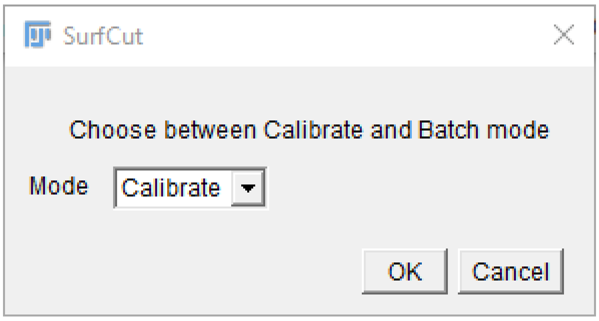

The macro has two modes: the first one, called "Calibrate", is to be used in order to manually find the proper settings for the signal layer extraction, but can also be used to process samples manually one by one. The second one, called "Batch", can then be used to run batch signal layer extraction on series of equivalent Z-stacks, using appropriate parameters as determined with the "Calibrate" mode. Go to section C. for the calibration mode and section D. for the batch mode.

C. Calibration mode

4. First, select the "Calibrate" mode and click OK.

5. A navigation window opens. Select the confocal stack that will be used for the calibration.

Remember that the stack should be in .tif and that the top of the stack should also be the top of the sample.
Note that in addition a log window will open. It will display information on the processing throughout the use of the macro.

6. The stack will open, and a pop-up window will ask you to define the input values for "Gaussian Blur" and "Threshold".

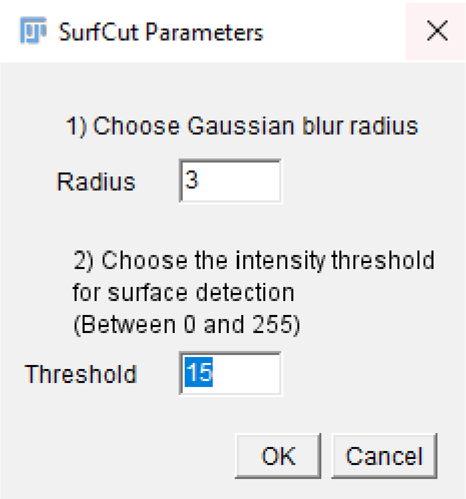

The Gaussian Blur is a mean to reduce the noise and smooth the signal. This is particularly important in order to obtain a smooth signal detection during the binarization of the signal. We found that a value of 3 µm works usually well for us. However, if your signal is very heterogeneous, e.g. for cortical microtubules, a higher value can help homogenize the signal and obtain a good surface detection.

The threshold value then depends on the intensity of the signal in your stack.
You can start with the pre-set values and check the result

7. Click on "OK" to initiate the image processing: (1) Gaussian Blur, (2) signal binarization, (3) edge detection, and (4) output binarized image and 3D visualization of the output image in the 3D Viewer.

   Note that you can follow the processing of the images in the log window.

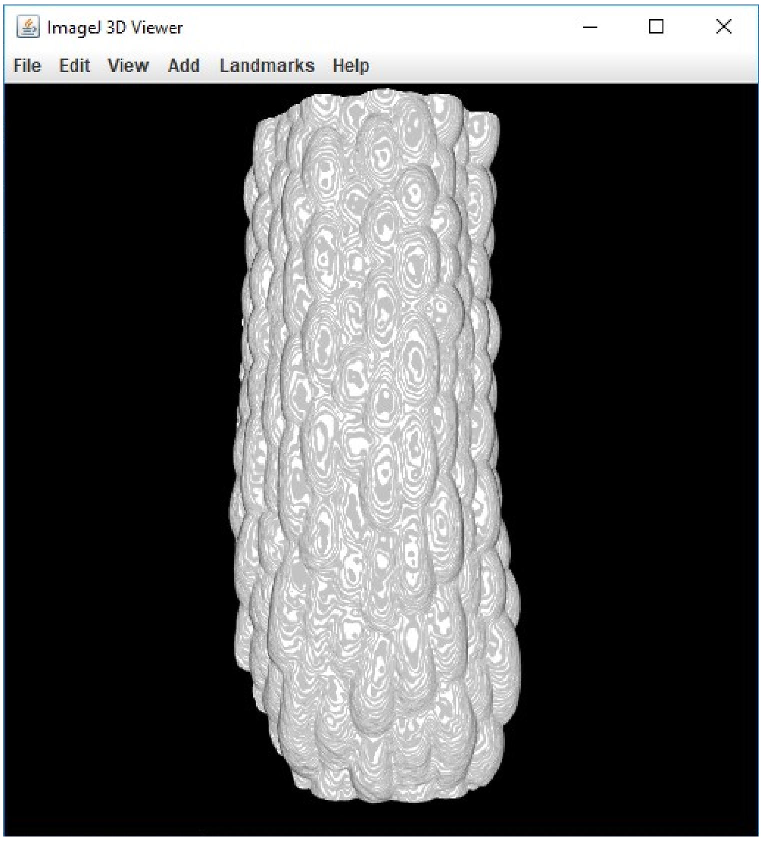

8. A new pop-up window will prompt you to check the quality of the edge detection.

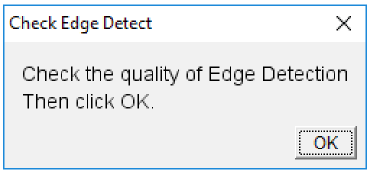

While this window is open you can freely interact with the 3D viewer in order to zoom-in and rotate the sample to check if the detected surface is smooth, homogeneous and presents no holes.

Below, on the left, is an example in which the Gaussian Blur was too low and the threshold too high. Holes in the detection of the surface indicates that the selected threshold was too low. Rough and irregular surfaces indicate that the radius of the Gaussian Blur is too low, while over-smoothed surface surfaces suggest that the radius is too high.

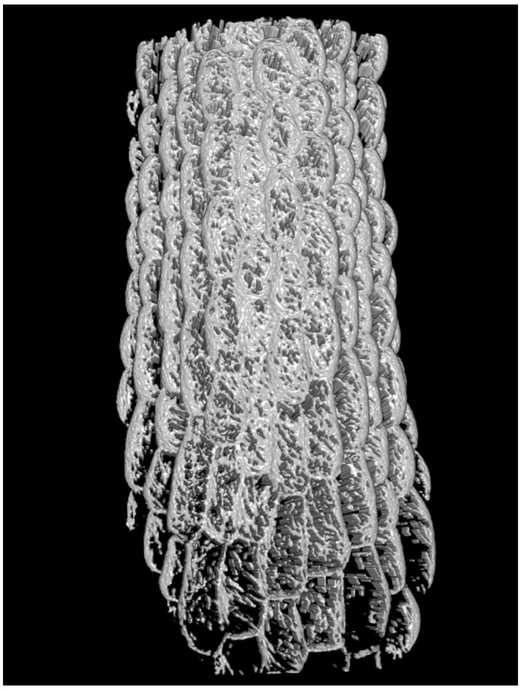

Bad: Many holes throughout the sample

Gaussian Blur = 1

Threshold = 80

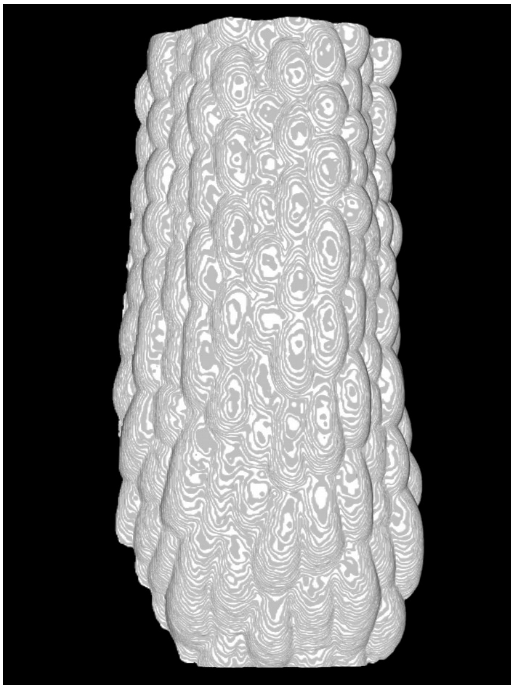

Good: Smooth continuous surface

Gaussian Blur = 3

Threshold = 15

9. Once you have checked the quality of the edge detection, click “OK” in the “Check Edge Detect” pop up window. A new pop up window will appear and ask you whether you are satisfied with the edge detection:

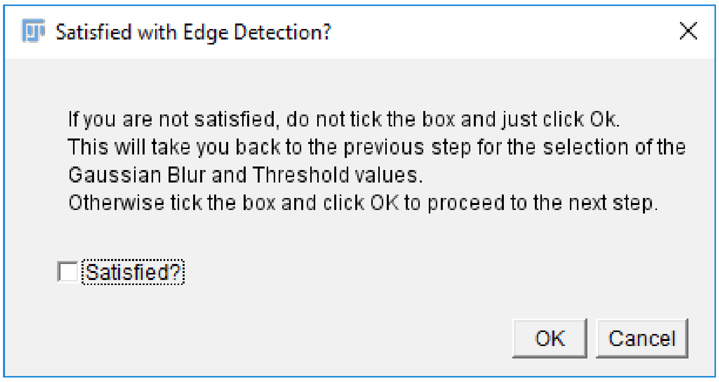

- If you are satisfied, tick the “Satisfied” box and click “OK”. This will take you to the next step.

- If you are not satisfied simply don’t tick the “Satisfied?” box and click “OK”. This will take you back to step 6, where you will be able to test new values for the Gaussian Blur and the Threshold. You can repeat this as many times as necessary until you find the proper settings for edge detection.

10. When you proceed to the next step, a pop-up window will ask you to choose the depths between which the stack will be cropped relative to the detected surface. This value is in micrometers. The voxel properties of your image in micrometers, are automatically filled based on the metadata of the image. If no data is found these values will all be set to 1. You can modify these values if necessary.

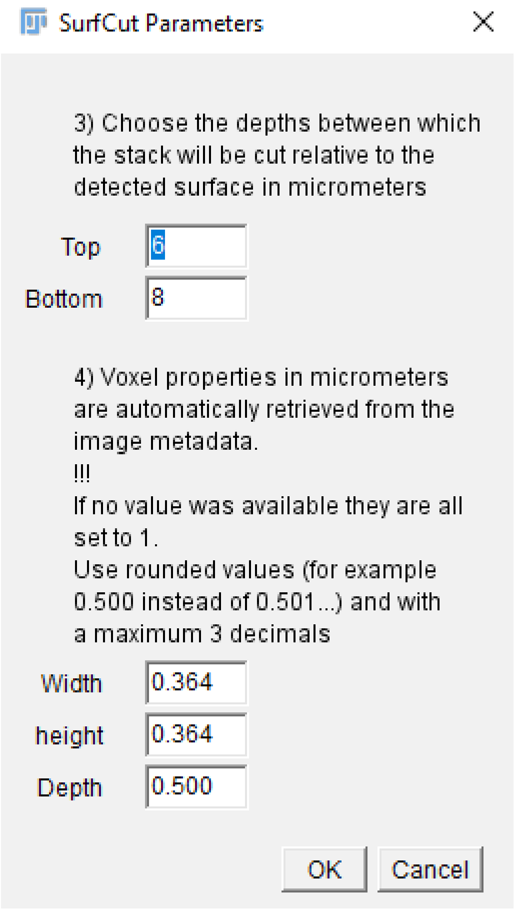

11. Once you have entered all the required values and pressed “OK”, the stack is processed, and the macro generates four images that can be useful to check the quality of the output:

- Two orthogonal reslices in the X and Y axis, through the center of the stack showing an overlap of the raw signal (green) and the cropped stack (thin white line; The thickness of this line depends on the thickness of the cropped signal layer)

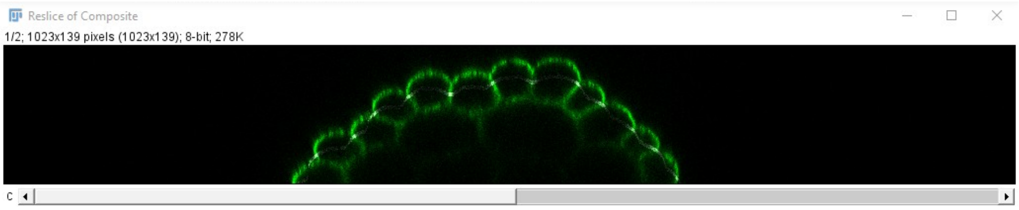

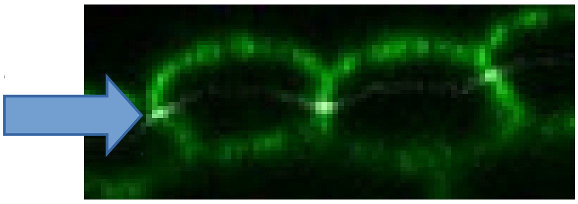

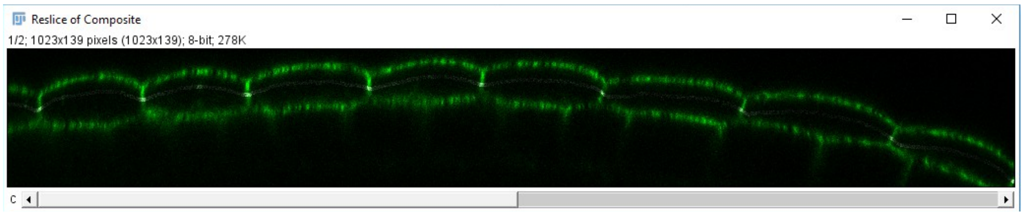

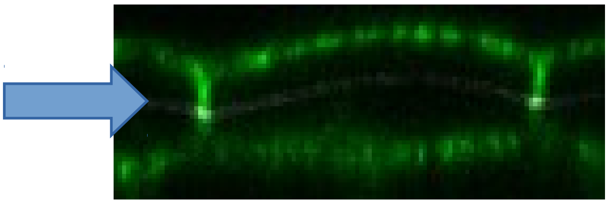

- The original stack max intensity projection

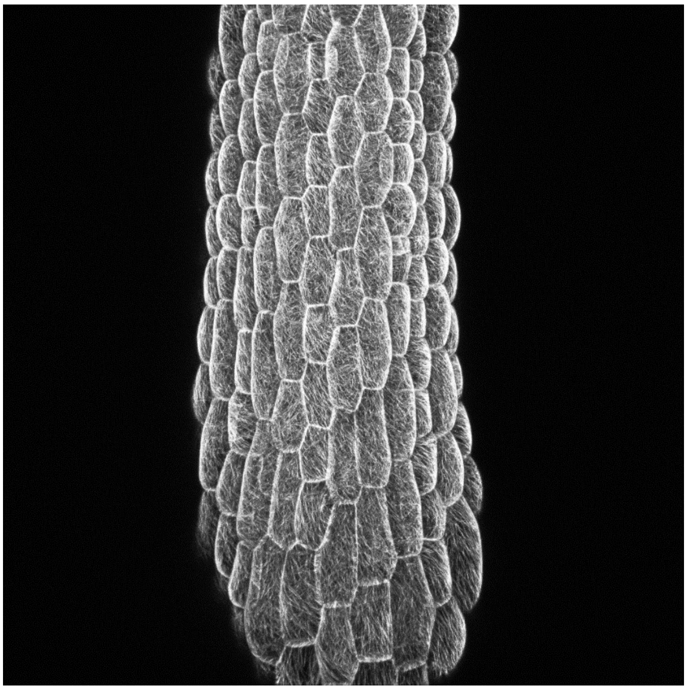

- And the SurfCut output max projection

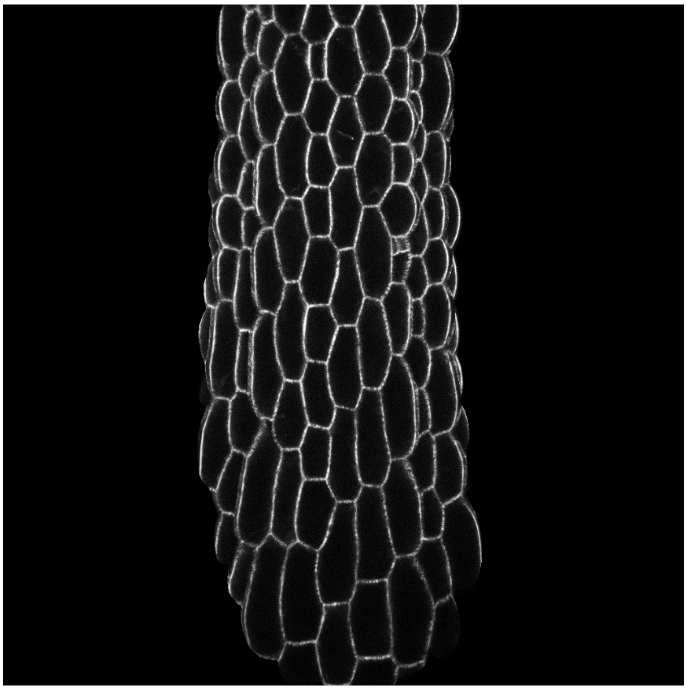

Note that by choosing the values for top and bottom depth of cropping you incidentally choose the thickness of the cropped stack. Although usually the thinner layers output nicer results, you cannot use values lower that the Z interval between the slices of your stack.

12. Similarly to step 8, a pop-up window will prompt you to check the quality of the SurfCut output. While this window is open you can freely interact with the different images generated in order to check the quality of the output and determine whether it matches with your expectations of signal layer extraction.

13. Similarly to step 9, once you have checked the quality of the SurfCut output, click “OK” in the “Check SurfCut Result” pop up window. A new pop up window will appear and ask you whether you are satisfied with the SurfCut Output. You can repeat this step as many times as necessary until you find the proper settings.

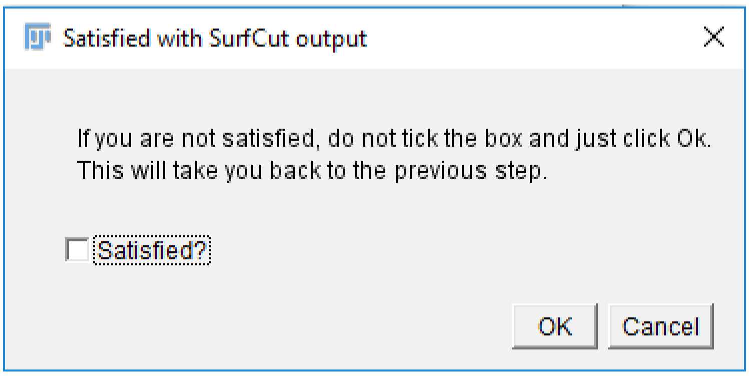

14. If the cropped image is satisfying, you will then choose which files will be saved. You can add a suffix to the files to specify some characteristic of the parameter that you calibrated. e.g for cell contour of the L1 layer, outer cortical signal (see exemple in the next part).

    You can choose to save the parameter file, containing all the parameters you have used to obtain the final SurfCut projection of this calibration step. You can also save the SurfCut projection as well as the original whole stack projection for comparison.

    Then click OK.

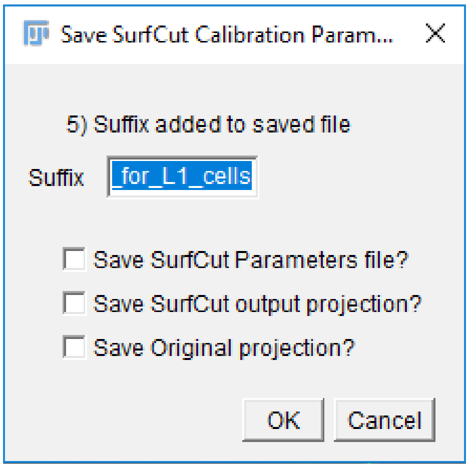

15. At the end of this calibration step, you can find a folder named “SurfCutCalibrate” in the same directory as your confocal stack. This folder contains the files saved in step 14.

16. The macro will then ask you whether you are done or if you want to process additional image stacks. If you select “No” the macro will stop. If you select yes you will be taken back to the beginning of the macro where you can process additional images manually (in Calibration mode) or run a batch analysis.

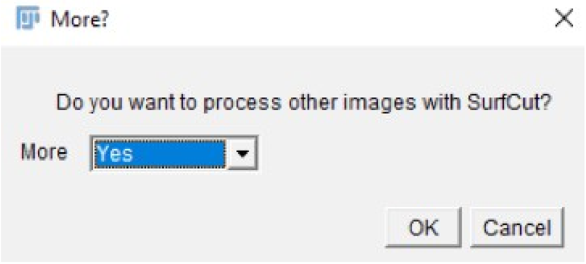

D. Running the script in batch mode

17. This time choose the “Batch” mode and click OK.

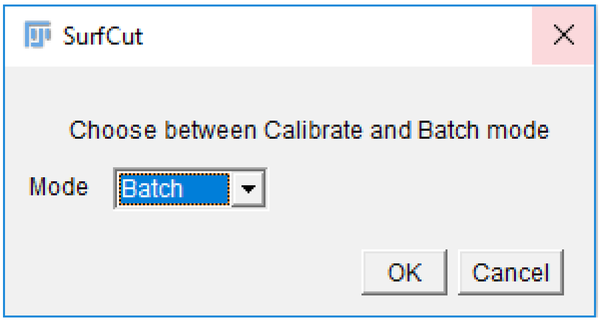

18. A navigation window will appear. From this window select the folder containing the confocal stacks that you want to process in batch with SurfCut.

Note that it is preferable that this folder contains only the confocal stacks that you want to process with SurfCut. Remember that the stacks should be in .tif and that the top of the stack should also be the top of the sample.

19. A pop-up window will ask you to choose whether you want to load a parameter file or if you prefer to enter the parameter manually. If you load a parameter file made during the Calibration process described above, all the parameters will be pre-filled in the next step.

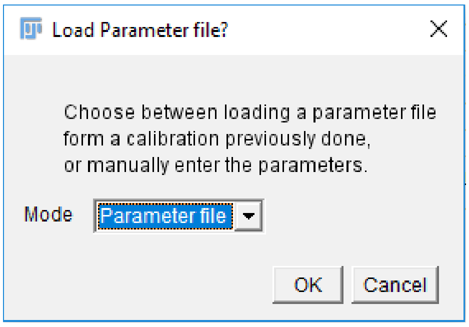

20. You can then further modify the parameters if necessary.

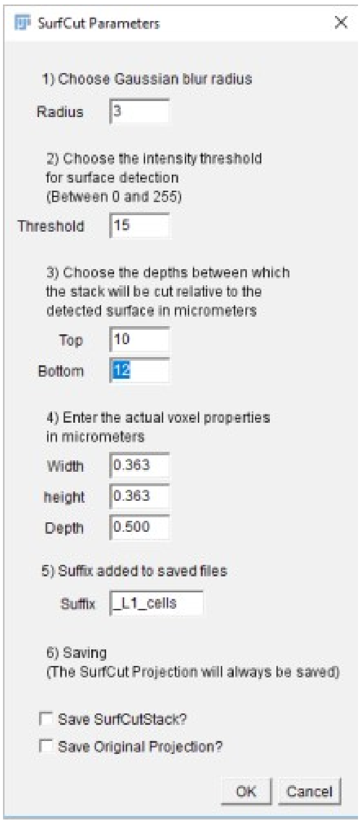

The SurfCut projections generated will be automatically saved, but you can also choose to save the SurfCut stack (3D stack of the signal layer extracted) if you wish to keep the 3D information, as well as the projection of the whole original stack, for comparison.
Then click ok to run the batch processing.

Note that you can follow the processing in the log window.

21. The SurfCut macro creates a results folder in the working directory defined in step 3, named "SurfCutResults". It contains all the saved output (e.g SurfCut projection, SurfCut stack, Original projection) as .tif files, as well as a .txt file containing the input parameters and a log of the analysis.

Application example:

Semi-automated high-throughput cortical microtubule array analysis.

Because the method that we describe is a signal layer extraction method, it is not only limited to the extraction of cell contours and can, for instance, extend to extract outer epidermal cortex signal as well. This is particularly useful in the case of cortical microtubule (CMTs) organisation quantification, because multiple layers of CMT arrays overlap in a classical 2D projection, which affects the proper analysis of this signal. In addition, because the confocal signal acquired is anisotropic (different resolution in Z compared to X,Y) in many cases it remains preferable to carry out analyses in 2D (with a 2D projected image) in order to avoid such bias.

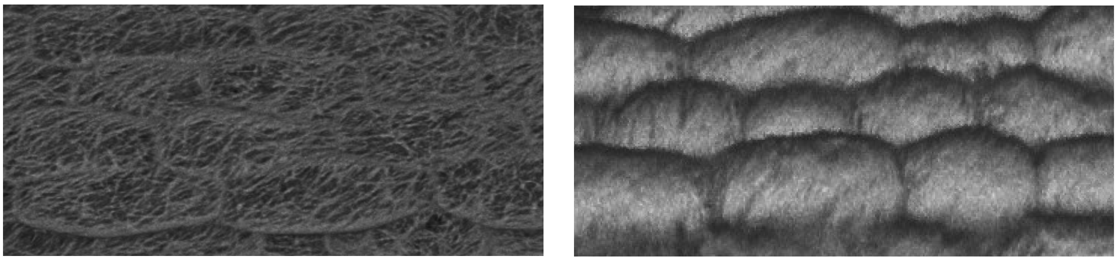

Left is the x,y view (from the top in Fiji 3D viewer) while right is the y,z view (from the side), showing the anisotropic nature of the confocal signal in epidermal cells of Arabidopsis thaliana hypocotyl expressing a marker for microtubules.

Here we describe the use of a pipeline that we have designed to semi-automatically quantify CMT arrays from 3D confocal stacks. The part of this pipeline after the SurfCut signal extraction (part C and D below) has already been reported in [17] in which signal layer extraction (part B) was performed manually with MorphoGraphX [25].
Briefly, (1) we use SurfCut to specifically extract the cell contour signal as well as the outer epidermal CMT signal. (2) We use the cell contour signal to create ROIs for each cell in each sample. (3) These ROIs are applied to the extracted CMT signal and CMT arrays are analysed with an automated version of FibrilTool [29,33].

These three processes correspond to three successive Fiji macros ("SurfCut.ijm", "Segmentation4FTBatch.ijm" and "FibrilTool_Batch.ijm"; see prerequisites). Each of these macros can be used to process batch samples, making the analysis high-throughput.

A. Prerequisites

- Fiji (https://fiji.sc).
- The "SurfCut.ijm" macro file (https://github.com/sverger/SurfCut).
- The "Segmentation4FTBatch.ijm" macro file (https://github.com/sverger/Segmentation4FTBatch).
- The "FibrilTool_BatchSeg.ijm" macro file (https://github.com/sverger/FibrilTool_Batch).

- The imageJ "MorphoLibJ" library (for the segmentation in "Segmentation4FTBatch.ijm") [30]. See installation procedure at https://imagej.net/MorphoLibJ.
- Data: A folder containing 3D confocal stacks in .tif format, in which the top of the stack should also be the top of the sample (example data available at https://github.com/sverger/SurfCut/test_File and http://doi.org/10.5281/zenodo.2577053).

B. Signal layer extraction with SurfCut

1. The first step is to extract both the cell contour and the outer epidermal CMT signal for all your images using SurfCut. You should first define the proper parameters for both type of extraction with one of your stacks, using the "Calibrate" mode.

For instance, in the images below, we have cropped the signal at a distance of 10 µm to 12 µm from the surface to extract the cell contours (left), and at a distance of 1 µm to 3 µm from the surface to extract the outer epidermal cortex (right).

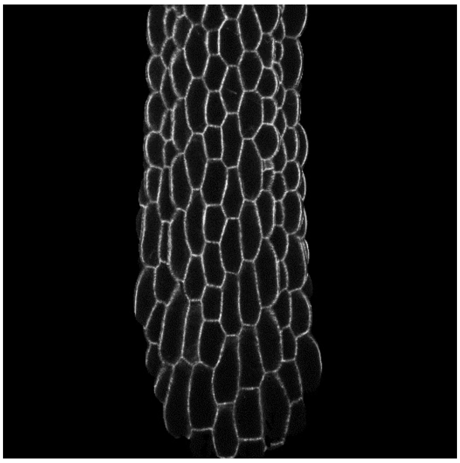

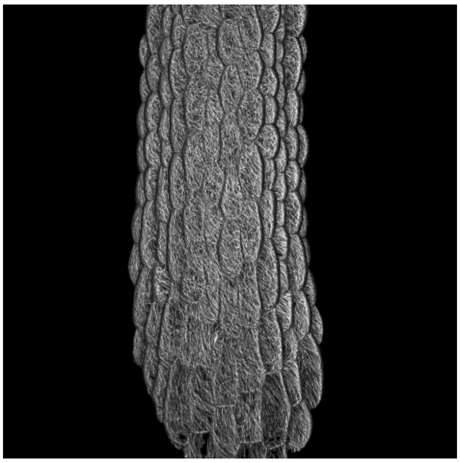

2. Once you have adequate parameters, first run the batch signal extraction for the cell contours.

*Note: It is important at this step to add in the SurfCut parameter window, a suffix for your images finishing with "**_cells**".*

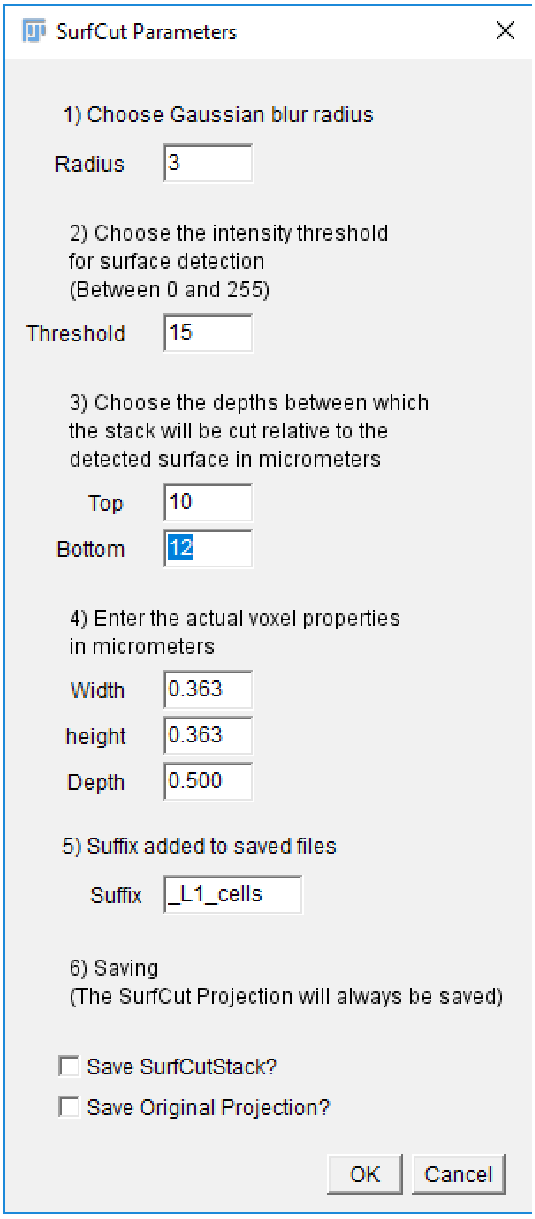

Also do not save the SurfCut stack nor the original projection.

3. Run the same batch signal extraction for the outer epidermal CMT signal, using the proper parameters, and this time using the suffix "_MTs".

4. The "SurfCutResult" folder should now contain for each stack, a 2D cell contour projection (e.g. SampleName_1_cells.tif, SampleName_2_cells.tif, ...) and a 2D outer epidermal CMT projection (e.g. SampleName_1_MTs.tif, SampleName_2_MTs.tif, …), as well as the parameter files for both signal extractions.

Note that it is critical for the following macros to work that the suffix of these images be respectively …_cells.tif and …_MTs.tif.

C. Segmentation and ROIs creation

1. Run the “Segmentation4FTBatch.ijm” macro.

2. A navigation window will open. From this window select the “SurfCutResult” folder created in the previous step (B.).

3. You will then have to process each image semi-automatically: First a Gaussian Blur of Radius 3 microns is automatically applied, and the image is opened with the Morphological segmentation tool of the MorphoLibJ library. A segmentation is run automatically with the default parameters (Watershed Segmentation Tolerance = 10).

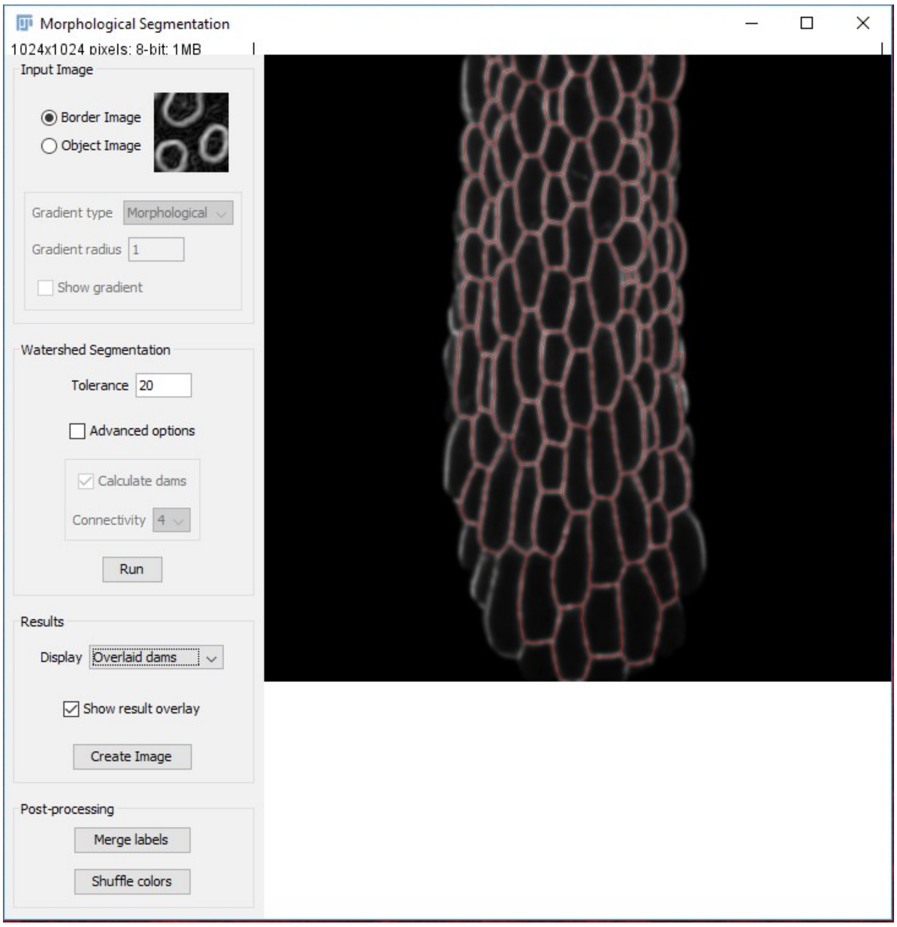

4. The automatic segmentation in step 3 may not be adequate (over- or under-segmentation). Check the segmentation and if necessary, re-run it. You can empirically find the best “Watershed Segmentation Tolerance” value (see image above in the red rectangle, in this case the value was changed to 20) and re-run the segmentation as many times as necessary. When you are satisfied click “OK” in the window below.

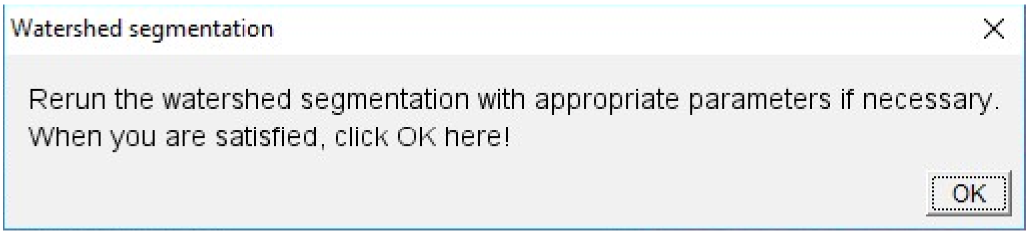

5. Next an outline of the segmentation (watershed lines) is generated and is automatically dilated four times. This is to exclude some of the signal from the cell contours (that can be remaining in the CMT image) that could bias the CMT array analysis. The Fiji "Analyse Particles..." function is then automatically run on this dilated segmented cell contour image, showing the identified ROIs as an overlay as well as in the ROI manager.

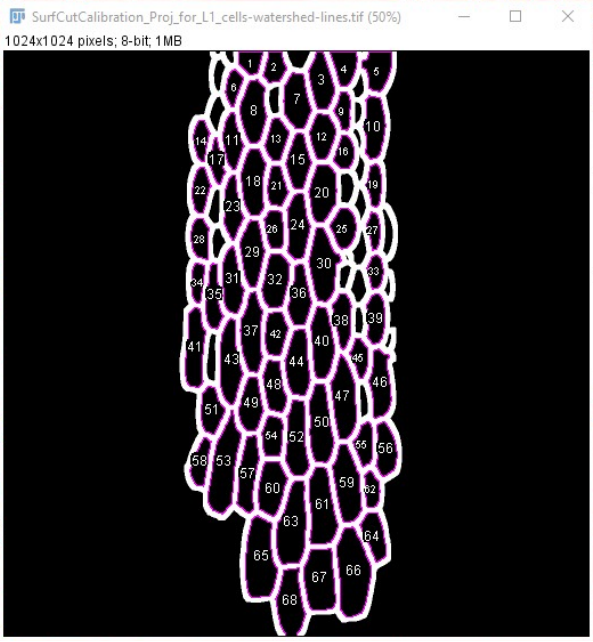

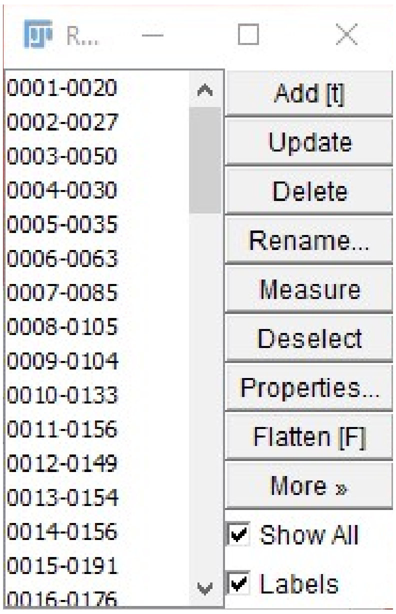

7. The "Analyse particles..." function is run by default with the particle size parameter at 1000-100000 pixels. This is to filter out the background as well as the smaller cells that would be meaningless for the analysis (too few pixels to analyse for FibrilTool). However, this may depend on your analysis and the nature of your images. If you are not satisfied with this parameter, you can un-tick the "Satisfied" box and click "OK". This will take you to the "Analyse particles..." parameter window, where you can set your preferred parameters and re-run the "Analyse Particles..." function. This can be repeated as many times as necessary.

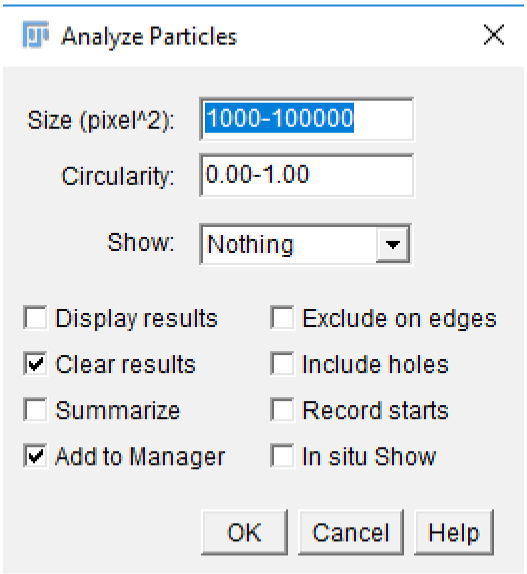

8. Next the generated ROIs are overlaid on the corresponding CMT image. At this step you can check whether the ROIs are properly positioned.

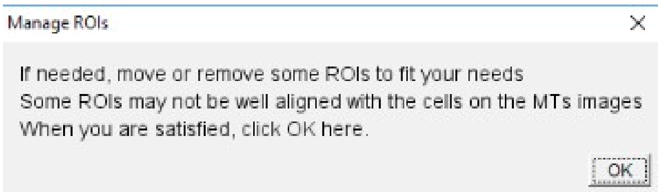

ROIs can be moved, deleted (see centre image) and created (see right image, blue ROI). You can for instance delete ROIs which are too much on the side of a curved sample since their analysis will be less meaningful. You can select individual ROIs by clicking on their label number, delete them with the backspace or del key, and create new ROIs with the Fiji "Polygon selections" tool.

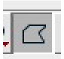

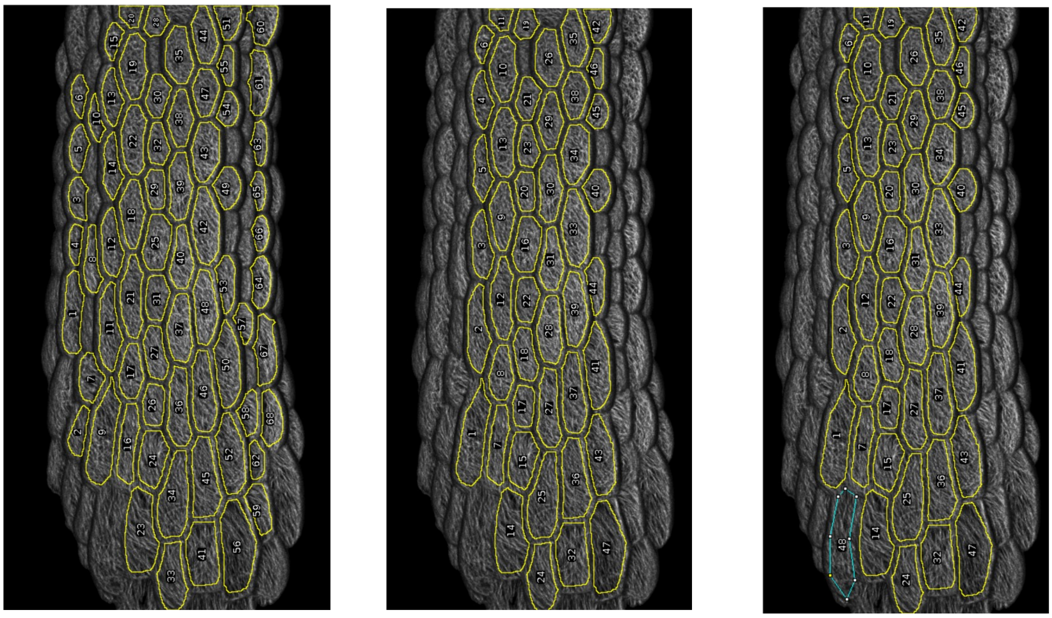

The closed polygon can then be added to the ROI list by pressing Ctrl + T.

9. When satisfied with your ROI set click “OK” in the “Manage ROIs” window. This will save the ROI set in a .zip file with the suffix “..._MTs_RoiSet.zip” and proceed to the next sample.

10. After processing all the samples, the “SurfCutResult” folder should now contain for each stack, an additional ROI set file (e.g. SampleName_1_MTs_RoiSet.zip, SampleName_2_MTs_RoiSet.zip, …).

D. FibrilTool Batch

Note that before running FibrilTool batch you should make sure that the log window is empty or close it. This will avoid pre-existing text in the log window to be saved in .txt output files of FibrilTool.

1. Run the “FibrilTool_Batch.ijm” macro.

2. A navigation window will open. From this window select the “SurfCutResult” folder created in step (B.).

3. Choose the channel in the pop-up window. Click “OK”.

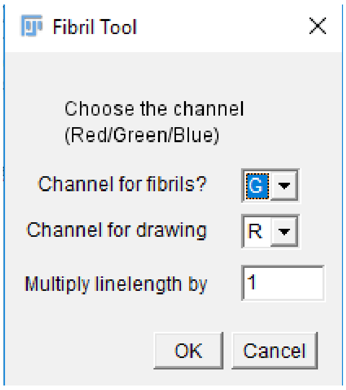

4. The macro will process all the ROIs of all the samples in the selected folder automatically and for each sample, save the FibrilTool output (red lines) overlaid on the CMTs in a .tif image with the suffix “..._MTs-MTs.tif”, as well as a “..._Log.txt file containing the values of anisotropy and principal angle of the CMT array for each ROI (see [29,33]) for the details of FibrilTool file output).

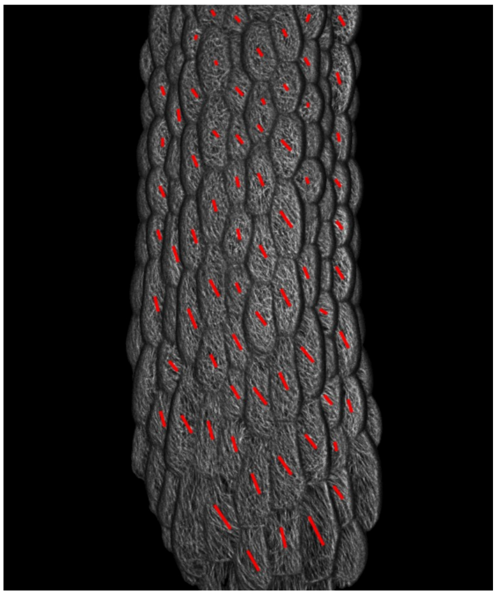

References: See main text
